# Cooling down with Entresto. Can sacubitril/valsartan combination enhance browning more than coldness?

**DOI:** 10.1186/s13098-022-00944-4

**Published:** 2022-11-23

**Authors:** Marina Nikolic, Jovana Novakovic, Galina Ramenskaya, Vladimir Kokorekin, Nevena Jeremic, Vladimir Jakovljevic

**Affiliations:** 1 Department of Physiology, Faculty of Medical Sciences, University of Kragujevac, Kragujevac, Serbia; 2 Department of Pharmacy, Faculty of Medical Sciences, University of Kragujevac, Kragujevac, Serbia; 3 First Moscow State Medical University IM Sechenov, Moscow, Russia; 4 Department of Human Pathology, First Moscow State Medical University IM Sechenov, Moscow, Russia

**Keywords:** Sacubitril, Valsartan, Cold, Obesity

## Abstract

**Background:**

It is a growing importance to induce a new treatment approach to encourage weight loss but also to improve maintenance of lost weight. It has been shown that promotion of brown adipose tissue (BAT) function or acquisition of BAT characteristics in white adipose tissue (terms referred as “browning”) can be protective against obesity.

**Main text:**

Amongst numerous established environmental influences on BAT activity, cold exposure is the best interested technique due to its not only effects on of BAT depots in proliferation process but also de novo differentiation of precursor cells via β-adrenergic receptor activation. A novel combination drug, sacubitril/valsartan, has been shown to be more efficient in reducing cardiovascular events and heart failure readmission compared to conventional therapy. Also, this combination of drugs increases the postprandial lipid oxidation contributing to energy expenditure, promotes lipolysis in adipocytes and reduces body weight. To date, there is no research examining potential of combined sacubitril/valsartan use to promote browning or mechanisms in the basis of this thermogenic process.

**Conclusion:**

Due to the pronounced effects of cold and sacubitril/valsartan treatment on function and metabolism of BAT, the primary goal of further research should focused on investigation of the synergistic effects of the sacubitril/valsartan treatment at low temperature environmental conditions.

## Background

Prolonged imbalance between energy intake and expenditure is the main cause of the global pandemic of obesity. Driven by this chronic impaired balance and excessive fat accumulation in the body, obesity has profound impact on tissue insulin sensitivity affecting systemic glucose homeostasis. Therefore, people with obesity are prone to develop variety of diseases including type 2 diabetes mellitus (T2DM), cardiovascular diseases and metabolic syndrome [1–3]. It is considered that imbalance between decreased physical activity, high energy carrying foods and inappropriate central appetite suppression are related to development of obesity [4]. Hence, there is a growing interest and importance to induce a new treatment approach that will encourage weight loss but also improve maintenance of lost weight. In this line, different methods of obesity treatment have been used to control or decrease body weight. Majority of them are primarily based either on reducing calorie intake through pharmacological approaches and diets, or on increasing energy expenditure through increased physical activity. However, mostly developed drugs are designed to target food intake through central appetite suppression which is reversible process. Consequently, the search for the safe and efficient way to increase energy expenditure by the manipulation of peripheral mechanisms has become to be important not only to overcome the weight regaining but also to diminish the side effects of drugs or other interventions [3, 5, 6].

Since the energy surplus is accumulated in the form of adipose tissue in the body, this tissue has become the central focus of the studies which are interesting to evaluate the mechanisms involved in obesity-related diseases [7, 8]. First, it has been considered that adipose tissue is a passive storage for the excess calories. Afterwards, it was known to be an effective endocrine organ that releases many adipokines as well as free fatty acids (FFA), which lead further effect on the other tissues such as brain, liver, and muscle to regulate energy balance, food intake, and insulin sensitivity [4]. Adipose tissue is primarily divided into two types of fat depots with different biological functions known as white adipose tissue (WAT) and brown adipose tissue (BAT). However, a third and novel type of adipose tissue has been recently discovered and deemed as bright/beige adipocytes. Exactly, beige adipocyte is brown-like cell that appears in WAT store [9]. WAT is primary site for lipid storage and mobilization, while BAT is thermogenic tissue responsible for heat production. Maintenance the balance between WAT and BAT are crucial to preserve energy homeostasis, while the accumulation of WAT leads to cardiovascular and metabolic disease complications [10]. In some experimental studies it has been shown that promotion of brown fat function or acquisition of BAT characteristics in WAT (terms referred as “browning”) can be protective against obesity and associated metabolic diseases [11–13]. While WAT distribution highly contributes to increased risk of metabolic diseases, BAT possesses adipocytes that use glucose and FFA as fuel indicating that this type of adipose tissue is in negative correlation with body fat percentage, body mass index, blood glucose levels and diabetes status in a opposite manner to WAT [14–17]. The main stimuli that activate BAT to use circulating FFA that are produced by catabolic lipid metabolism of WAT and produce thermogenesis is exposure to low temperatures. This process occurs via mediation of the uncoupling protein-1 (UCP-1) that is presented in the inner mitochondrial membrane of brown adipocytes. Further, this protein dissipiates the proton gradient that creates the respiratory chain as heat. Moreover, exposure to low temperatures not only activates BAT but also induces appearance of UCP-1 positive adipocytes in WAT which can be considered as a strong stimuli for transition of WAT into BAT [18].

In this review we aimed to outline the current understanding of cold induced thermogenesis, other mechanisms of WAT browning and summarize important markers associated with BAT promotion. Also, we focused to highlight the mimic of the cold induced thermogenesis through various pharmacological agents and its application in therapy of metabolic diseases.

## Main text

### Adipose tissue: structure, types and anatomy

The adipocytes in the human body can be generally divided into two main anatomical depots, subcutaneous adipose tissue (SAT) and visceral adipose tissue (VAT). While SAT is the tissue located under the skin, VAT is lining the internal organs and can also be additional classified based on its anatomical location. In that sense, VAT is divided in intrathoracic, abdominal, and etc., but intrathoracic VAT is further classified as epicardial adipose tissue (EAT) and pericardial adipose tissue [19–21]. There are considerable differences in endocrine function, lipolytic activity, and insulin sensitivity between SAT and VAT. Many investigators established a close correlation between adverse metabolic profile and VAT accumulation, as opposed to even ameliorative profile of SAT. In addition to closely association to insulin resistance, VAT is also related to exaggerated inflammatory state, which explains close relationship between VAT accumulation and cardiometabolic risk and morbidity [19, 22, 23].

**Fig. 1:**
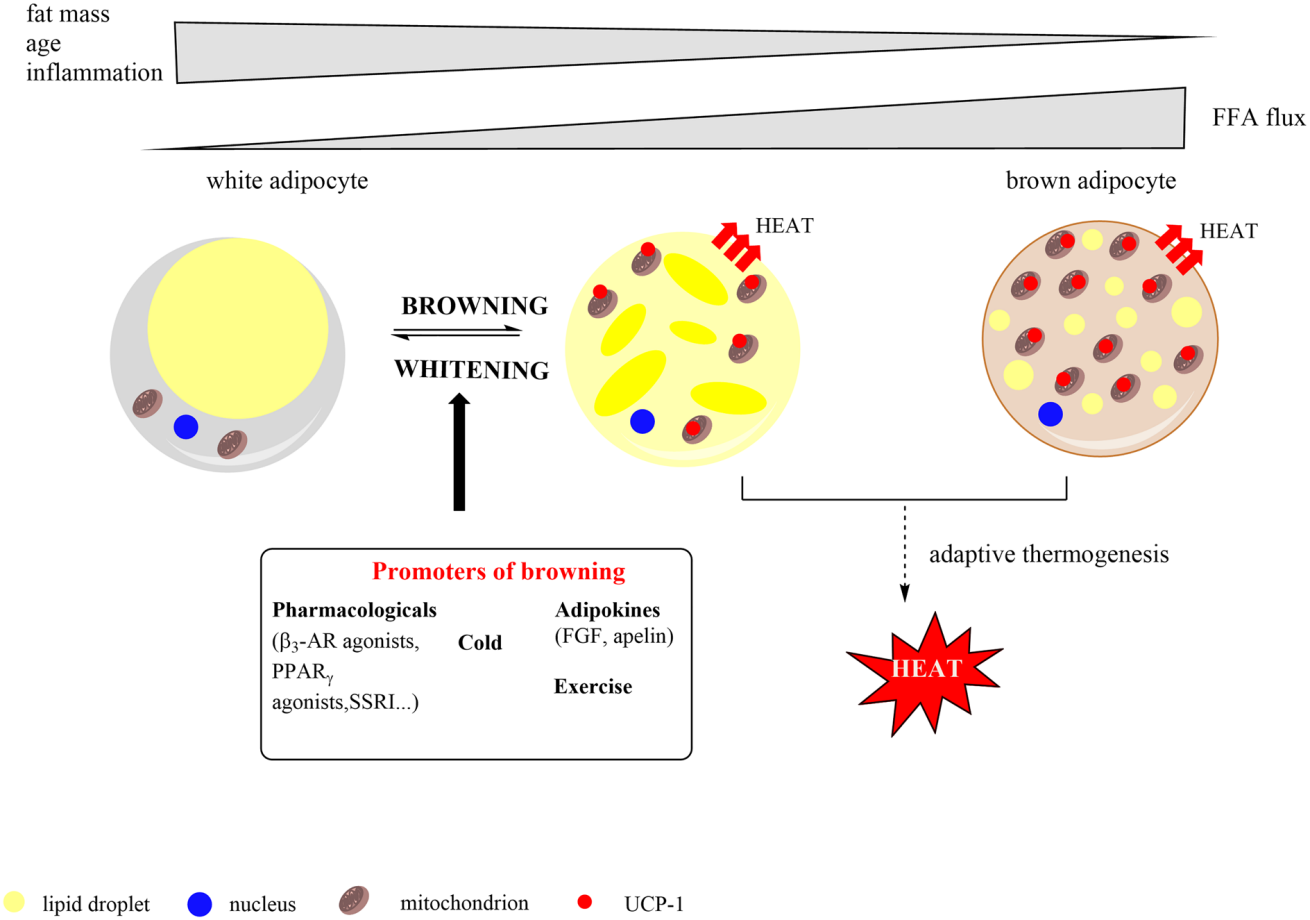
Different types of adipocytes and examples of agents that promote browning of white adipocyte cells. *FFA* free fatty acid, *PPARγ* peroxisome proliferator-activated receptor gamma, *SSRI* selective serotonin re-uptake inhibitor, *FGF* fibroblast growth factor

According to its functional role, phenotype and profile of gene expression, adipose tissue can be further divided by two as WAT and BAT [24]. Significant differences between WAT and BAT through the morphological, biochemical, and physiological characteristics were well described to the date. White adipocytes are distinguished by spherical shape, thin, rare, and elongated mitochondrial, which size mainly depends on single lipid droplet presented in them. This lipid droplet is composed of triglycerides that occupied more than 90% of the cell volume [9, 25, 26]. Moreover, WAT is the main organ of numerous products secretion including hormones, growth factors, enzymes, cytokines, complement factors, and matrix proteins making this tissue responsible for the regulation of healthy fat mass arrangement and maintenance of adequate balance between food intake and energy expenditure (Fig. 1) [27]. On the other hand, brown adipocytes are characterised with multilocular lipid droplet structure which means that triglycerides are located in smaller and multiple vacuoles. The most typical organelle for BAT is mitochondria, which consist of numerous, spherical, large, and packed with laminar cristae. The brown colour of BAT is attributable to its great vascularisation and high mitochondrial density. Moreover, there is evidence to indicate how BAT promotes a 10–20% level high contribution in energy expenditure comparing to basal metabolic rate. Because of this greater oxygen requirement, BAT contains denser neural supply than WAT. Nonetheless, being a key component, a densely packed UCP-1 in BAT mitochondria, provides difference between BAT and WAT and the main BAT`s physiological function of thermogenesis [28, 29]. In addition to the classical WAT and BAT, different stimuli induce browning of WAT into the beige adipose tissue that is specially recognized by interspread population of brown-like adipocytes [30]. From the morphological aspect, beige cells are characterized by multilocular lipid droplet, high mitochondrial content, and expression of a basic set of BAT specific genes [31]. Although beige adipose tissue has potential to undergo thermogenesis as a common ability of BAT, there are some differences in characteristics of these two similar adipose tissues. Firstly, it refers to different embryonic precursors reliable for brown adipocytes rise and distinguished gene structures [32–34]. While BAT expresses extremely high UPC-1 levels under basal conditions as well as other thermogenic genes, beige cells show this genes expression only in case of the response to activation of ß-adrenergic receptor [12]. However, there is unclear whether brown and beige cells have similar functions? Even if a recent study fully suggested how these adipose tissues express the comparable amounts of UCP-1 and consequently the similar thermogenic capacities [33], it is needed to have further studies to get a proper answer to it. The regulators that induce “beigeing” or “browning” of WAT will be discussed widely in the following paragraphs.

The principal function of WAT as a most represented adipose tissue mass in humans is to store vast amounts of nutrient in lipids and then realise them as FFA when food is scarce [35]. On contrary, BAT accounts less than 1% of total body weight and dissipates the energy of stored lipid droplets as a heat in a process called “nonshivering thermogenesis”. The thermogenic process in BAT occurs via UCP-1 that uncouples the mitochondrial proton gradient, generated during oxidative phosphorylation, from adenosine triphosphate (ATP) production. Namely, in eukaryotic cells, energy is stored in mitochondria as a proton gradient across the inner mitochondrial membrane and just that energy is used for synthesis of ATP through the enzyme ATP synthase through the protons flowing down to their concentration gradient. In conditions when protons return along the gradient without creating ATP, the stored energy is wasted as heat through UCP-1. This process is demonstrated by immunohistochemistry analysis as marker of BAT [26, 36]. However, there is abundant evidence revealing the mechanisms that control UCP1-dependent thermogenesis. Briefly, thermogenesis begins by norepinephrine realising from the terminal sympathetic neurons ends that innervates adipose tissue, which leads to β3-adrenergic receptors (β3-AR) activation. Further, the adenylate cyclase is activated which consequently increases intracellular cyclic adenosine monophosphate (cAMP) levels and activates protein kinase A (PKA). In this signal cascade, PKA further phosphorylates substrates such as lipid droplet-associated protein perilipin, p38-mitogen-activated protein kinase (p38-MAPK), and hormone sensitive lipase (HSL), leading to mobilization of FFA from lipid droplets to activate UCP-1 [30]. Consequently, a numerous of harboured highly oxidative, naturally uncoupled mitochondria actively oxidize metabolic substrate to facilitate heat production [37, 38]. While this process is thought to be a main contributor to non-shivering thermogenesis, recently published studies proposed that some portions of non-shivering thermogenesis is independent of UCP-1 activity or even BAT [39–41].

### Age-dependent adipose tissue in body

Brown fat is found in abundance in small mammals who are predisposed to temperature loss due to small body volume/body surface ratio. Since long period the presence of active BAT is restricted to new-borns and infants, presumably to provide warmth in the cold environment they encounter at birth. Although adult humans are largely devoid of brown fat, BAT is located in the several regions including cervical, supraclavicular, axillary, paravertebral, mediastinal, and upper abdominal regions. It is proposed that distribution of brown fat serve as a warming mechanism for the blood supply to vital organs [24, 26, 42]. BAT volume and presence increase rapidly during puberty and reach a pick at the adolescence [43, 44]. Notably, BAT depots rapidly decrease with age, metabolic related diseases, hypertrophy, and reduced tissue sensitivity to β adrenergic stimuli. Even though BAT is considered negligible in adults, its enhanced reserves were found in subjects exposed to cold climates for a long time (as experienced by Scandinavian outdoor workers) or in patients with pheochromocytoma. It was observed that repetitive exposure to mild cold prevents the obesity development and that is in close correlation with increased BAT activation [7, 42, 45]. Thus, maintaining metabolically active BAT depots and browning capacity throughout life may contribute to healthier metabolic phenotype in the elderly.

 Given the context of numerous aforementioned favorable functions of BAT, it has been easily overlooked the importance of recruiting brown adipocytes as a cell type with ability to utilize the excess FFA, facilitate weight loss and improve metabolic health.

## Transcriptional networks and beige adipogenesis

Functionally, cell differentiation can be considered as a change in gene expression patterns so that transcriptional events trigger preadipocyte differentiation (adipogenesis) and adipocyte function. An elaborate network of the transcription factors encodes hundreds of proteins that are responsible for the control of transcription [46]. Among them, two master of adipogenesis regulation are master process, named as peroxisome proliferator-activated receptor gamma (PPARγ) and CCAAT/enhancer-binding protein α (C/EBPα). Without PPARγ, precursor cells are unable to express any known aspect of adipocyte phenotype indicating that this regulator is necessary for in vitro and in vivo adipocyte formation and function. Moreover, ectopic expression of PPARγ is crucial for adipocytes differentiation in muscle cells and in fibroblasts. On the other hand, C/EBPα is not essential for brown adipocyte differentiation but plays a significant role in cellular insulin sensitivity [47, 48]. Looking from the mechanistic level, PPARγ closely interacts with the basic leucine-zipper factor C/EBPα and thus regulates genes expression that are specific for adipocytesdifferentiation. The transcriptional cascade set in first wave which refers to expression of proadipogenic or antiadipogenic transcriptional cofactors, C/EBPβ and C/EBPδ. After just a few hours of adipocytes stimuli, these cofactors can bind to DNA or chromatin and interact with the adipogenic transcription factor glucocorticoid receptor (GR) which is expressed in the early adipocyte differentiation and regulates cell cycle and cell growth genes. This first wave activates the second wave in this transcription cascade which is in touch with PPARγ and C/EBPα activation. Therefore, the actions of PPARγ and C/EBPα can be modulated by large set of proadipogenic or antiadipogenic transcriptional cofactors, but the role of many of them in WAT browning remains not clear yet. [29, 48, 49].

### Regulation of UCP-1 expression

Brown adipocytes express a wide spectrum of so-called “thermogenic genes” that control both browning and metabolic function of these cells. Among them, UCP-1 is crucial for the function of brown and beige adipocytes and only a few identified UCP-1 transcription factors were identified as specific for these adipocytes [29, 50]. The main regulation of UCP-1 expression is mediated by sympathetic tone through ß-adrenergic signalling which couples to enzyme adenylyl cyclase, responsible for conversion of ATP to cyclic cAMP which further phosphorylates PKA and activates p38 mitogen-activated protein kinase (p38-MAPK). This signalling pathway mediates phosphorylation of many transcriptional factors involving cAMP—response element binding protein (CREB) which regulates the expression of type 2 iodothyronine deiodinase (DIO2). Afterwards, this enzyme catalyses the conversion of inactive tetraiodothyronine (T4) to triiodothyronine (T3) which directly activates UCP-1 gene transcription regulation [51, 52]. Moreover, the half site cAMP– response element (CRE) interacts with both CREB and nuclear factor activating transcription factor 2 (ATF2). The phosphorylation of ATF2 is mediated by p38-MAPK pathway which in turn initiates UCP-1 transcription through its CREs [53]. The second target of p38-MAPK is the PPARγ—coactivator 1-α (PGC-1α), which associated with PPARγ binds to the UCP-1 promoter and thus drives transcription of the UCP-1 gene [51, 53]. In addition to mentioned positive regulatory elements, it is noteworthy to emphasise the significance of the repressors of UCP-1 gene transcription. It was reported that receptor-interacting protein 140 (RIP140) interacts with nuclear receptors, binding to UCP-1 gene enhancer region and thus contributes to maintenance of UCP-1 transcriptional repression in brown non-differentiated pre-adipocytes. However, there are recent reports indicating negative regulation of human UCP-1 gene transcription via unliganded vitamin D receptor (VDR) [51].

### Role of 12,13-Dihydroxy-9Z-octadecenoic acid (12,13-diHOME) in browning

12,13-diHOME was originally identified as a class of lipids released from adipose tissue that acts as a signalling mediator, thereby affecting metabolic health [54]. Also, this batokine is recognized as oxylipin, a category of bioactive lipids obtained from polyunsaturated FFA [55]. Hence, biosynthesis of 12,13-diHOME as well as its isomer 9,10-diHOME originates from linoleic fatty acid (ω6 FA), which is metabolized by cytochrome P450 and results in the formation of 12,13- or 9,10-epOME epoxides. Further, these compounds are hydrolysed by soluble epoxide hydrolases (sEHs) and finally create diols 12,13-diHOME and 9,10-diHOME [56]. Four different sEH-encoding genes have been known so far: *Ephx1* and *Ephx2* are two major isoforms found in the adipose tissue [57]. It was observed that expression and activity of sEHs are positively regulated during adipogenesis implying the significant role of 12,13-diHOME in adipocyte`s maturation [58]. Given the fact that under 12,13-diHOME WAT acquire typical characteristics of BAT, this lipokine is appeared as “thermogenic metabolite” [54]. This thermogenic function of 12,13-diHOME is supported by the fact that in response to cold stimuli or exercise BAT releases this batokine further leading to regulation of BAT fuel uptake. Likely, under chronic cold environment BAT increased circulating levels of 12,13-diHOME via enhanced lipolysis and expression of *Ephx1* and *Ephx2* thus initiating its biosynthesis. Partially, 12,13-diHOME through autocrine-paracrine mechanism initiates translocation of FFA transporters in brown adipocytes resulting in improved FA uptake and decreased circulating levels of triglycerides. The final step of this cascade is facilitation of thermogenesis by supplying a fuel [54, 56].

Although there is not enough data to examine pharmacological induction of 12,13-diHOME expression, it was reported that treatment with β3-adrenergic agonist increased *Ephx1* in subcutaneous WAT and *Ephx2* expression in BAT after 10 days treatment. This suggests that circulating levels of 12,13-diHOME might be increased in response to stimulation of β3-AR. Based on this fact, it can be assumed that cold exposure increases 12,13-diHOME expression triggering release of norepinephrine from sympathetic nerve system leading to activation of β3-AR and increasing this lipokine expression [56].

### PGC-1α-PPAR complex mediated regulation

PGC-1α is early induced in BAT differentiation and it is dominantly expressed in mature brown cells in comparison with white adipocytes. Furthermore, PGC-1α expression is rapidly and highly induced by cold stimuli and turns on several key components of the adaptive thermogenesis. This process further makes it the most important regulatory protein in thermogenesis [59]. The expression of PGC-1α is substantial for cAMP-mediated induction of UCP-1 gene transcription as its expression is activated following phosphorylation by the cAMP-PKA-p38/MAPK signalling pathway. Also, PGC-1α binds to a number of nuclear hormone receptors including PPARγ, PPARα and retinoid X receptor (RXR), and induces the overexpression of UCP-1. Hence, treating with agonists of these receptors leads to increased UCP-1 transcription [60, 61]. It is previously observed that overexpression of PGC-1α in adipocytes resulted in increased oxygen consumption and promotion of mitochondrial biogenesis. Moreover, thermogenic machinery is failed to be efficiently activated in response to adrenergic stimulation in brown fat cells that have scarce level of PGC-1α indicating that appropriate PGC-1α expression is necessary for the acute transcriptional activation of thermogenesis but not for brown cells development per se [62].

### PRDM-16 mediated activation

The transcriptional factor PRDM16 (PR domain zinc finger protein 16) is considered as a master controller of brown and beige cell identity via playing an important role in regulation of differentiation-linked brown gene program. Actually, PRDM16 binds to gene enhancers of brown adipocytes and helps to establish promote interactions with transcription factors such as peroxisome proliferator-activated receptor alpha (PPARα) and PPARγ, and coregulators (PGC-1α). Indeed, the activity of PGC-1a–PPAR complex is modulated by PRDM16 which expression contributes to enhanced UCP-1 levels. Although PRDM16 is highly enriched in brown cells compared to WAT, its expression in white cells suppresses classic white adipocyte genes and leads to high UCP-1 level in inguinal WAT. This finding emphasizes the significant role of PRDM16 in browning of WAT [63, 64]. However, it is known that some cofactors might block the expression of PRDM16. Overexpression of transducin-like enhancer of split 3 (TLE3) specifically inhibits the PRDM16–PPARγ interaction in adipocytes tissue thus promoting lipid storage while its deletions, vice versa induces transcription of genes involved in thermogenesis [65].

### AMPK–SIRT1–PGC‑1α pathway

Promotion of browning also can be reached through deacetylation of PPAR by NAD-dependent protein deacetylase sirtuin 1 (SIRT1) which mimics the effects of chronic activation of PPAR through direct activation of PGC-1α [29]. Indeed, different compounds activate 5`-AMP-activated protein kinase (AMPK) consequently leading to activation of SIRT1. Indeed, AMPK is well-recognized energy sensor taking an important place in the regulation of cellular homeostasis [66–68]. Activation of AMPK is crucial not only for promotion of thermogenesis in BAT but also in white adipocytes. The balance of evidence suggests that AMPK-mediated activation of SIRT1 leads to signalling cascades highly involving expression of PGC-1α and PPARγ as well as to upregulation of UCP-1 in fat cells. In addition, AMPK can regulate adipogenesis throughout various pathways including the inhibition of mTOR pathway, demethylation of PRDM16, activation of PPARγ. Recent data had shown that AMPK is also essential for β-adrenergic-mediated browning. Concluding, activation of AMPK is considerable regulator of browning process of beige cells [69, 70].

### Fibroblast growth factor (FGF) in browning

The expression of FGF family is enhanced in response to some environmental stimuli including exercise training or cold exposure. Interestingly, FGF6 and FGF9 are considered as potential inductors of UCP-1 expression in brown and white adipocytes which is independent of adipogenic differentiation. Actually, stimulation of prostaglandin E_2_ (PGE_2_) biosynthesis induces FGF6- and FGF9-mediated UCP-1 expression showing that this pathway of browning is completely unrelated to conventional adipogenic transcription factors. Moreover, it was observed that FGF21 as a member of the FGF family is realised from skeletal muscle and promotes browning via stabilization of PGC-1α protein without affecting the expression of its transcriptional genes. In the fed state, FGF21 regulates PPAR activity in white cells through feed-forward loop thereby controlling the adipogenesis in WAT [71, 72].

## Cold BAT activation

Amongst numerous established environmental influences on BAT activity, cold exposure is the best studied stimulus for BAT activation, since the primary role of brown adipocytes is to mediate non-shivering thermogenesis. Also, cold exposure is by far the strongest symphatogenic signal triggering norepinephrine release from sympathetic nerve fibers. Afterwards, this catecholamine activates the β-adrenergic receptor and finally stimulates cAMP-dependent pathway [3, 73, 74]. Furthermore, cold exposure affects not only the expansion of BAT depots in proliferation process but also de novo differentiation of precursor cells via β-adrenergic receptor activation [75]. It is interestingly to note that prolonged cold exposure induces high UCP-1 expression in white adipocyte depots with special affinity to inguinal WAT as a main subcutaneous depot in rodents which is highly sensitive to browning even in mild stimulation environment. Reversely, epididymal WAT depot of male mice is sufficiently resistant to browning wherefore inguinal white cells are usually used in studies examine beige fat development [76].

In condition of adrenergic stimulation removal (warm temperatures), beige cells lose their expression of UCP-1 and accept a morphology that is characterised for WAT. However, these adipocytes may be encouraged to recover their multilocular morphology and restore UCP-1 expression in response to another cold adaptation. Comprehensively, the thermogenic phenotype of beige tissue is reversible indicating that continuously adrenergic stimulation is necessary in order to maintain the thermogenic profile of these adipocytes [77, 78].

Adipocyte fate in response to cold exposure is also investigated from the immunological aspect. This environment induces activation of eosinophils in adipocytes, boosts interleukin (IL)-4 and IL-13 levels and causes the alternative activation of M2 macrophages. The activated macrophages further amplify the secretion of catecholamines and thereby intensify browning of white cells and brown adipocytes thermogenesis [79, 80]. Furthermore, an increase in macrophage infiltration, especially M2 macrophages, is observed in patients and mice with burns [79]. This type of macrophages in the burned subcutaneous white adipocytes undergoes alternative activation to induce tyrosine hydroxylase expression accordingly stimulate catecholamine production. So, M2 macrophages with anti-inflammatory effects provide WAT browning, energy expenditure and systemic thermogenesis [2, 79].

Aforementioned activated cAMP-signalling pathway induces phosphorylation of PKA modifying lipid droplet binding proteins and hormone sensitive lipases. This cascade leads to FFA release, glycerol, and glutamate into the circulation which later serve as fuel sources for thermogenesis induction [81]. In addition, FFA initiates UCP-1 activation but also possesses ability to regulate adipocytes differentiation in WAT and BAT through PGC-1α-PPAR complex-mediates pathway. Studies conducted on rodents showed that acclimatisation to lower temperature led to significant increase in genes expression that are related to FFA synthesis, FFA uptake and oxidation in brown fat cells [82, 83]. Moreover, clinical studies concluded that BAT activation is in correlation to cold-induced lipolysis, enhanced FFA oxidation and re-esterification as well as to energy expenditure in either skinny or peoples with obesity [84, 85]. This implies that activation of brown adipocytes by cold exposure increase FFA uptake and metabolism in BAT. Altogether they may have favorable effects on obesity and patients with T2DM.

Content of many fuel sources glucose seems to play the most important role and acts as substantial contributor in BAT homeostasis [82]. By increasing the expression of glucose transporter protein 4 (GLUT4), either acute (4-48 h) or chronic (10 days) cold exposure enhances glucose uptake and insulin sensitivity. It was shown that glucose uptake is also intensified in BAT of people with obesity, glucose intolerant individual. Considering the fact that glucose uptake under the cold is more prominent in brown fat cell compared to other tissues (WAT, skeletal muscle, brain, liver, and heart), this strategy is observed as a capable stimulus to increase insulin sensitivity in BAT [82, 86]. Moreover, previously conducted studies have shown that activated glucose uptake is up 12-fold greater after cold exposure in comparison to insulin-stimulated glucose uptake in BAT. These powerful effects of cold exposure on BAT glucose uptake are association with improved whole-body glucose disposal and insulin sensitivity in healthy or adults with obesity, or even in patients with T2DM [87–89]. A recent study, on healthy individuals exposed to cold, demonstrated that the most of the cold-induced cold uptake is interfered with deeper and central muscle of the neck and back, and the muscles at the inner thigh. These results pointed out the coordination between brown fat cells and muscle response to low temperatures [90].

In addition, cAMP-mediated signalling pathway takes a certain place in cold-exposure induced realise of FGF21. Actually, this growth factor increases energy expenditure and core temperature likely via induced FFA catabolism and perhaps through thermogenic activation of brown adipocytes [91]. Liver-derived FGF21 is capable of activating thermogenesis in neonatal brown fat cells, and it is highly expressed in adults BAT [92]. A study examining the circulating levels of FGF21 showed that mild cold exposure increased the level of this transcriptional factor of browning which is associated with intensified BAT activation. However, even plasma level of FGF21 was increased, the circulating source of FGF21 was not observed. Altogether, these findings highlight that cold exposure may increase FGF21 level, and utilization of FFA and glucose, but FGF21 is not required for the maintenance of metabolic homeostasis in cold environment [93–95].

## Pharmacological promoters of browning

Following the document, the biology and origin of BAT development, by a detailed review of transcription factors and pathways involved in browning in previous sections, we will further focus on pharmacological agents that favour the acquisition of brown adipocytes–like characteristics in WAT but also emphasizing their proposed mechanisms of action.

### Selective β-3 adrenergic receptor agonists

Activation of thermogenesis in fat cells mediated by selective activation of β_3_-AR has been reported in earlier rodent models. Dating back to 1992 years, Cousin et al. demonstrated significant increase in UCP-1 expression in WAT depots as in typical brown fat in rats submitted to treatment with β_3_-AR agonist [96]. In another pioneering study, group of scientists also reported that chronic treatment with selective agonist of β_3_-AR increased UCP-1 content three- to fourfold in interscapular BAT. In addition, this drug not only induced BAT proliferation and differentiation, and energy expenditure but also slowed development of WAT in the early stage of obesity [97]. Moreover, therapy with these drugs led to brown adipocytes occurrence within traditional WAT depots such as inguinal, epididymal, retroperitoneal and mesenteric fat depots [98]. The significance of β_3_-AR in activation of thermogenesis in adipose tissue exposed to cold is well documented in several studies. Indeed, it was shown that absence of these receptors in WAT led to failed response to cold, induction of “thermogenic” genes, and appearance of beige adipocytes. On the other hand, the lost appearance of β_3_-AR in BAT did not affect the adaptive response to cold exposure [99]. Hence, there is an absolute requirement for intact β_3_-AR signalling for cold-induced browning. A vast number of selective β_3_-AR agonists underwent clinical trials, but most of them were rejected due to lack of efficiency in activation of human β_3_-AR. However, the first tested β3-AR agonist, L-796568, on people with obesity showed acute increase (8%) of energy expenditure with no side effects on cardiovascular system due to no action on β1-AR and β2-AR. Nonetheless, chronic treatment with this adrenergic agonist did not lead to powerful activation of BAT thermogenesis. Recently discovered selective β_3_-AR agonist, mirabegron, induces a remarkably increase in resting metabolic rate (13%) in young male. Although its favorable effects on adipose tissue, some evidence had linked the use of mirabegron with cardiovascular side effects. However, previously conducted clinical studies that focused on this adverse effect have shown that mirabegron has acceptable safety profile when it is used at therapeutical doses. Together, it seems that mirabegron can be a considered as great anti-obesity drug due high potential to upregulate UCP-1 expression [100–102].

### Antidiabetic drugs

It has been shown that activation of PPARγ by synthetic ligands thiazolidinediones (TZDs) leads to an appropriate glycemia management in diabetic patients, but also may induce conversion of white cells to BAT. Indeed, TZDs directly bind to and activate PPARγ promoter of either white or brown adipocytes. Further, these drugs induce increased expression of UCP-1 and other mitochondrial genes, biogenesis of mitochondria, and multilocularisation of adipocytes in white fat cells depots. It is proposed that TZDs as a fully agonists of PPARγ induce aforementioned browning effects via PGC-1α expression in both WAT and BAT [50, 103]. In connection with tissue recruitment, activation of PPARγ enlarges brown adipocyte potential to take up and store FFA which suggests that BAT has a crucial role in antidiabetic action of PPARγ agonists [104].

Recently, it has been shown that the dipeptidyl peptidase-4 (DPP-4) enzyme is considered as adipokine secreted excessively in obesity. Hence, use of DPP-4 inhibitors may be effective treatment to fight with metabolic impairments of obesity. Lately, linagliptin as a DPP-4 inhibitor has shown powerful potential to increase PGC-1α expression which is an essential step to the browning. Moreover, it is established that DPP-4 inhibitors activate PPARα as a pleiotropic effect which is followed by PGC-1α induced mitochondrial biogenesis, and enhanced UCP-1 expression in white adipocytes thus promoting its browning [105].

As a selective sodium-glucose cotransporters-2 (SGLT-2) inhibitor, empagliflozin enhances energy expenditure, and UCP-1 expression in either BAT or WAT. This suggests that empagliflozin promotes adipose tissue browning, controls the proton flow back into the mitochondrial matrix, and at a same time consumes energy as a heat instead of ATP synthesis [106]. It was known that FGF21 as a browning promoter is highly expressed in response to cold stimuli, but it also can activate β_3_-AR in WAT and promotes browning. Chronic treatment with empagliflozin increases FGF21 plasma and hepatic levels indicating that this growth factor can mediate modification of energy metabolism to fat utilization in response to SGLT-2 treatment [107]. Moreover, treatment with empagliflozin led to reduced M1 proinflammatory macrophage infiltration but increased anti-inflammatory M2 macrophage population in fat cells, subsequently intensifying browning of white adipocytes [5].

### Drugs affecting the serotoninergic system

Serotonin (5-HT) system through central modulation stimulates thermogenesis and recruits beige adipocytes in addition to well-known ability to modulate feeding behavior. Consequently, pharmacological agents that affect serotoninergic system may serve as powerful inductors of 5-HT mediated action on adipose tissue. Selective serotonin re-uptake inhibitor (SSRI) fluoxetine is widely prescribed antidepressant that reduces body weight via central modulatory mechanisms. A recent conducted study demonstrated that chronic fluoxetine use decreases both body weight and WAT mass, while increasing BAT depots and UCP-1 expression [108].

## Sacubitril/valsartan (Entresto®) and browning

Considering actual epidemiological scenario representing high prevalence of obesity, it becomes necessary to design a new approach to prevent and combat with pathology and comorbities as consequence of increased body mass. Given that nutritional education and promotion of physical activity have not fully succeeded as an adequate discipline is required, the activation of BAT or browning of white fat cells is indicated as a new access to treat obesity. Several studies recently have documented beneficial effects of numerous pharmacological agents that can exert antiobesity effects inducing thermogenesis through different signalling pathways. A novel combination drug, sacubitril/valsartan marked by Novartis under the name Entresto®, has been shown to be more efficient in reducing cardiovascular events and death, but also heart failure readmission compared to conventional angiotensin-converting enzyme (ACE) inhibition [109]. However, to date there is no clear data either examining potential of Entresto® to promote white adipocytes browning or underlying mechanisms of the basis of this thermogenic process. Because of that, we will further review the ability of Entresto® to induce browning from an individual Sacubitril or Valsartan potential to promote WAT to BAT conversion.

Sacubitril is formulated as a prodrug which is hydrolysed into the active neprilysin inhibitor LBQ657. Afterwards, by neprilysin inhibition LBQ657 blocks the degradation of natriuretic peptides (NPs) enabling promotion of their favorable effects including natriuresis and diuresis, aldosteron and renin inhibition, vasodilatation and reduction of blood pressure, protection of atherosclerosis, thrombosis, and so on [110]. Three different members of the NPs family exist, named as atrial natriuretic peptide (ANP), brain natriuretic peptide (BNP), and C-type natriuretic peptide (CNP). While ANP leads to reduction in blood pressure and cardiac hypertrophy through paracrine and endocrine signalling pathways, BNP locally prevents ventricular fibrosis. By contrast, CNP dominantly encourages long bone growth in addition to many other less known functions as well [111]. ANP and BNP are peptide hormones that are dominantly produced in the heart in response to pressure overload or heart failure, leading to the realization of their main hemodynamic action on cardiovascular system. These two hormones dominantly bind to natriuretic peptide receptor—A (NPR-A). On the other hand, natriuretic peptide receptor-B (NPR-B) is primary receptor of CNP and has related topology to NPR-A. The main function of natriuretic peptide receptor-C (NPR-C) is to clear NPs from circulation through the process of receptor-mediated internalization and degradation [111].

**Fig. 2:**
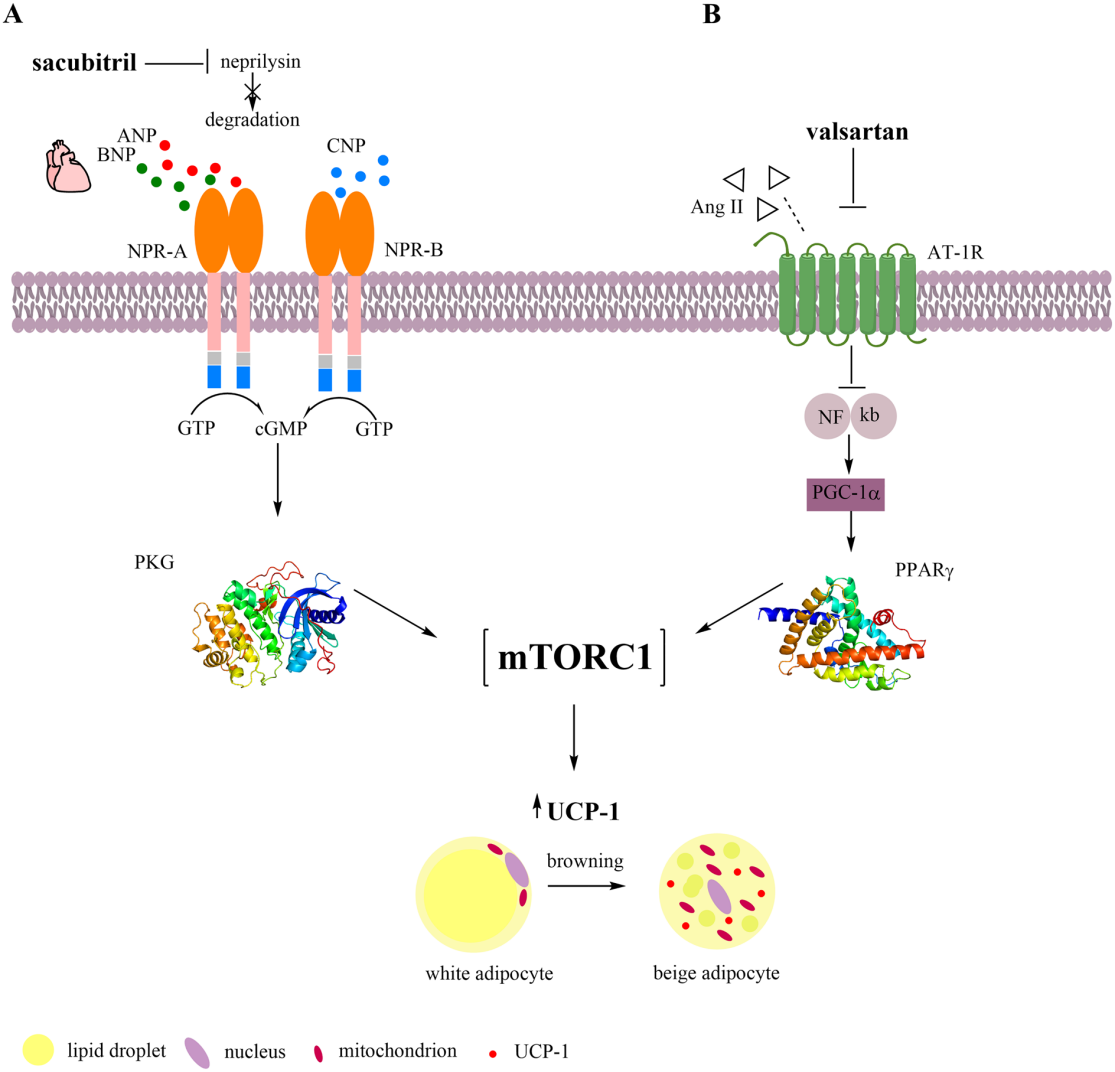
A proposed working model of **A** sacubitril and **B** valsartan browning action. *ANP* atrial natriuretic peptide, *BNP* brain natriuretic peptide, *CNP* C-type natriuretic peptide, *NPR-A* natriuretic peptide receptor-A, *NPR-B* natriuretic peptide receptor-B, *Ang II* angiotensin II, *AT-1R* type 1 angiotensin II receptor, *GTP* guanosine triphosphate, *cGMP* cyclic guanosine monophosphate, *PKG* protein kinase G, *NFκβ* nuclear factor kappa β, *PGC-1α* PPARγ-coactivator 1-α, *PPARγ* peroxisome proliferator-activated receptor gamma, *mTORC1* target of rapamycin complex 1; *UCP-1* uncoupling protein-1

**Fig. 3:**
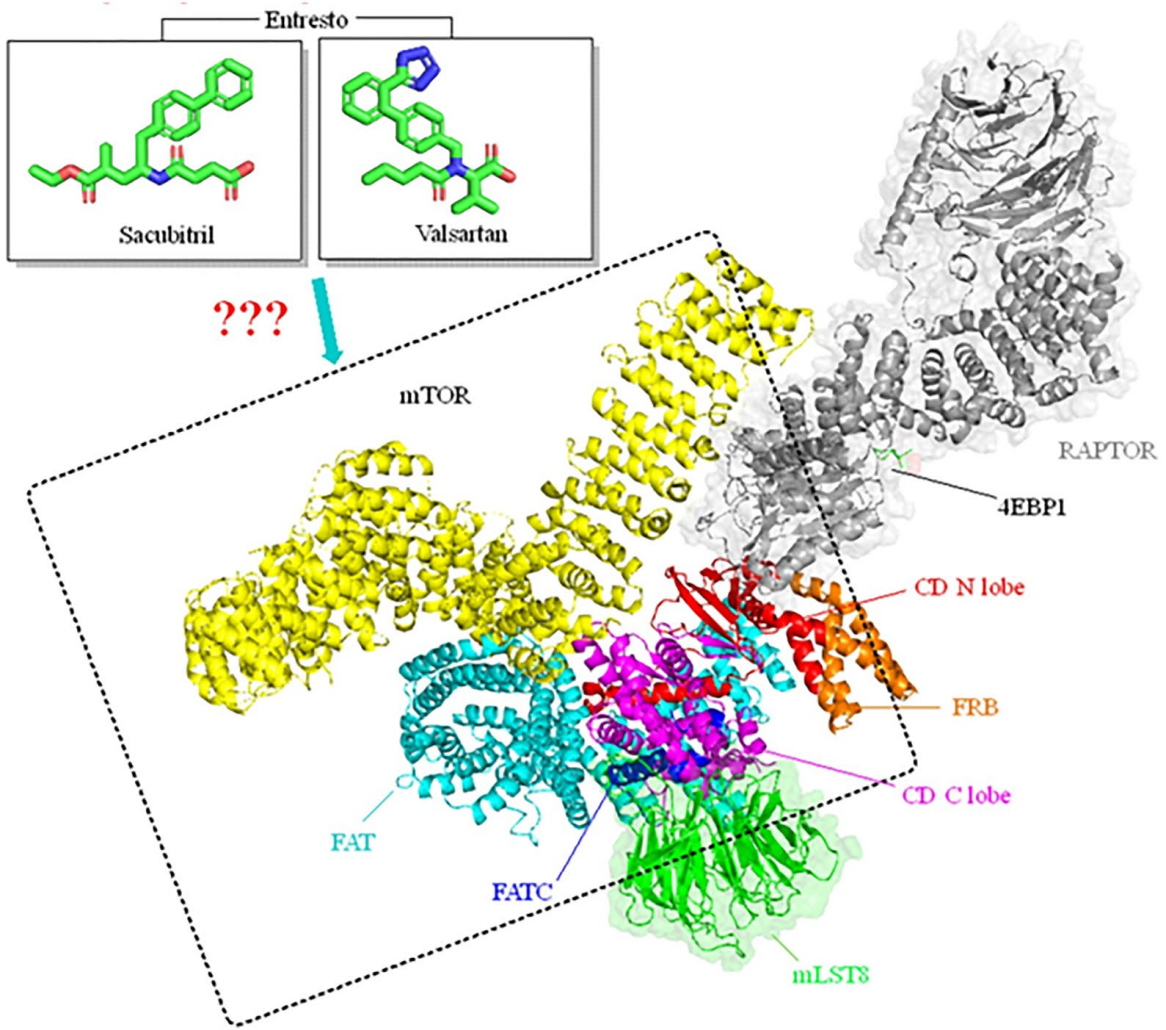
Schematic representation of sacubitril/valsartan binding to mTORC1. Structural domains of mTORC1 are presented in different colours: mLST-8 (mammalian lethal with Sec13 protein 8) is coloured green, RAPTOR (regulatory associated protein of mTOR) is coloured gray, serine/threonine-protein kinase mTOR is framed with dashed lines. 4EBP1 (Eukaryotic translation initiation factor 4E-binding protein 1) is presented in black letters. Structural domains of mTOR: FRB (rapamycin binding domain) is coloured orange, FAT (trans-activation domain-associated protein domain) is coloured cyan, FATC (C-terminal FAT domain) is coloured blue, while CD C and N lobe (catalytic domain) are coloured magenta and red

Binding of NPs to NPR-A results in generation of cyclic guanosine monophosphate (cGMP) from guanosine triphosphate, which further activates protein kinase G (PKG) and mammalian target of rapamycin (mTOR) targeting cells to perform effect (Fig. 2A) [5, 112, 113]. This atypical serin/threonine protein kinase, mTOR controls numerous cellular functions related to cell growth and homeostasis including protein synthesis, metabolism, survival, and proliferation [114]. Depending on which regulatory protein it binds to, mTOR can form two structurally and functionally distinct types of protein complexes [115]. Rapamycin sensitive mTORC1 is dimeric complex which is composed of mTOR and subunits RAPTOR (regulatory associated protein of mTOR) and mLST-8 (mammalian lethal with Sec13 protein 8) (Fig. 3) [116]. The molecular recognition mechanism between sacubitril/valsartan and mTORC-1 is still unknown, due to the lack of knowledge about simultaneously binding affinity of these drug/drugs to different subunits of mTORC-1.

In addition to favorable effects of NPs on hemodynamic regulation of renal and cardiovascular system, identification of natriuretic peptide receptors (NPRs) expression in adipose tissue attracted great attention to investigate ANP/BNP pathways in this tissue [5]. Growing evidence implicate that NPs also possess ability to regulate glucose homeostasis and energy balance via interorganic metabolic communication with NPRs expressed in adipose tissues [117]. Furthermore, it was observed that increased circulating levels of these peptides are in association with reduced body weight in human thus promoting lipolysis in human adipocytes [31]. Enhanced ANP level is linked to lipolysis induction in white adipocytes as well as thermogenesis in brown fat cells while both NPs through cGMP-PKG-mTORC1 pathway initiate higher UCP-1 expression and mitochondrial content in adipocytes. Together, the consequence of increased NPs levels through triggering the mTORC1 is activation of WAT browning and beige fat biogenesis (Fig. 2A) [117, 118]. The same results were obtained from the study conducted by *Bordicchia *et al. demonstrating that higher NPs concentrations are in positive correlation with promotion of beige adipocyte development in WAT but also with intensified gene expression in BAT. Exactly, this action is mediated through PKG that triggers lipolysis and induces browning using the similar mechanisms to activate transcriptional response in BAT. Specifically, PKG as well as PKA activates p38MAPK that further initiates the transcription of downstream thermogenic genes in nucleus of WAT such as UCP-1 and PGC-1α [31, 118, 119]. Additionally, a large number of experimental and clinical studies promote the attitudes that diabetes type 2, obesity and metabolic syndrome are diseases characterized by NPs deficiency, recognized as NPs handicap when adipose tissues are insensitive to circulating NPlevels. This statement formed on majority of evidence indicating that increase in NPs may lead to treatment of metabolic disturbance [120]. While intravenous infusion of ANP enhances the postprandial lipid oxidation and contributes to energy expenditure, it was shown that BNP infusion drive to positive metabolic profile decreasing ghrelin levels and hunger [120, 121]. In addition, it was observed that both mentioned NPs increased adiponectin synthesis by adipocyte and ameliorate glucose metabolism and insulin resistance through AMPK pathway. Therefore, intensifying adiponectin synthesis and reducing levels of proinflammatory cytokines IL-6 and tumor necrosis factor alpha (TNFα), NPs provide increased systemic insulin sensitivity [120].

The effects of NPs were also observed in experimental study indicating significantly higher WAT content in knockout NPR-A mice that suffer from cardiac hypertrophy compared to wild-type animas [118]. Apart from numerous endocrine factors that are involved in cGMP-PKG pathway as a catecholamine-independent pathway, the NPs concentrations could be significantly increased upon cold exposure. A recent in vitro study has shown that ANP cultured with brown adipocytes remarkably increased intracellular temperature in low-temperature condition through stimulation of p38-UCP-1 pathway [122]. The same results were confirmed by *Kimura *et al*.* indicating that ANP treatment of animals exposed to cold not only improved the HFD-induced insulin resistance but also promote browning of WAT due to ability of this NP to activate thermogenic gene expression. Moreover, those animals developed the tolerance to low-temperature exposure which emphasizes the adaptive heat-retaining potential of NPs in state of low body temperatures. Interestingly, systemic administration of ANP affects numerous adipose tissues but also hepatic tissue, resulting in diminished insulin resistance caused by HFD [117]. There are several possible explanations for increased NPs concentration under cold exposure, but it is assumed that in reaction to cold superficial blood vessels are contracted in an effort to prevent heat loss thus pumping the blood to the larger blood vessels and enhance NPs production in the heart [123]. In general, deeper examination of the mechanisms that are involved in NPs mediated adipose tissue browning either administrated synergistically with other drug or in conditions of lower temperatures are necessary given that NPs may serve as a great therapeutical tool to combat with obesity.

Valsartan, as an anti-hypertensive and anti-atherosclerotic drug is a member of selective and competitive type 1 angiotensin II receptor (AT-1) blockers (ARBs). In addition to common prescription of these drugs for treating cardiovascular disorders, blockers of rennin-angiotensin system (RAS) also reduce body mass and lead to better glycemic control in patients with obesity [124]. This is due to local presence of RAS in adipose tissues influencing adipocyte differentiation, adipokine secretion and transport of glucose [125]. Given that RAS component in plasma positively correlates with obesity, pharmacological manipulation of RAS activity may be a good approach to reduce adiposity. In that sense, AT-1 blockers have potential to reduce body mass and glycemic level with ability to increased expression of thermogenic genes and gain multilocular lipid appearance in white adipose tissue [126, 127]. In a recent study, it has been investigated in vitro and in vivo ability of losartan to promote browning via apelin induction. Apelin is as a member of adipokine family which promotes glucose uptake in skeletal muscle and adipose tissues, decrease insulin secretion and induce adipogenesis of brown fat cells. The study demonstrated the potential of losartan to induce browning of white adipocyte primarily via increased Akt/AMPK phosphorilation, but also through enhanced apelin expression [127]. Hence, it was shown that 5-week treatment with losartan induced thermogenic beige fat cells in subcutaneous WAT leading to reduced adiposity index and increased energy expenditure [124]. As AT-1 antagonists, sartans inhibit nuclear factor kappa β (NF-κβ) thus causing reduced gene expression of IL-6 and TNFα, but increase appearance of PGC-1α and consequently re-establish the activity of PPARγ. Related NF-κβ- PGC-1α-PPARγ pathway is likely to mediate in conversion of WAT into beige one after treatment with AT-1 blockers [128]. Moreover, antagonists of AT-1 receptors increase UCP-1 gene and protein expression in subcutaneous white adipocytes which coupled with various mitochondria thus formatting beige adipose tissue. Elevation in PPARγ activity after sartans treatment induces PRDM16 gene expression which maintains brown features in beige adipocytes. When PRDM16 expression is suppressed, beige fat cells switch to WAT which emphasize a decisive role of PRDM16 in keeping of beige cells phenotypes and their thermogenic potential [124, 128, 129]. Moreover, the central place of PPARγ in ARBs-mediated browning is also reflected through its activation by 9,10-diHOME, lipokine derived from ω6 FA, which appears to serve as a PPARγ ligand [130]. Although it is already known that PPARγ is one of the master regulators of adipogenesis, evidence suggest that ligand mediated PPARγ stimulation takes an important place in mTORC1 activation. Previous studies implicate the importance of crosstalk between PPARγ and mTORC1 in the regulation of adipose tissue metabolism. Exactly, it was shown that activation of mTORC1 via PPARγ leads to positive regulation of adiponectine production and secretion in subcutaneous fat cells. Moreover, it was observed that mTORC1 defficiency reduced tissue activities of PPARγ and C/EBPα as two main regulators of adipogenesis. However, up until know it remains unclear by which precise interaction PPARγ agonists activate mTORC1 in adipose tissue but what is certainly know is that this crosstalk does not involve increase in insulin sensitivity (Fig. 2B) [131].

**Fig. 4:**
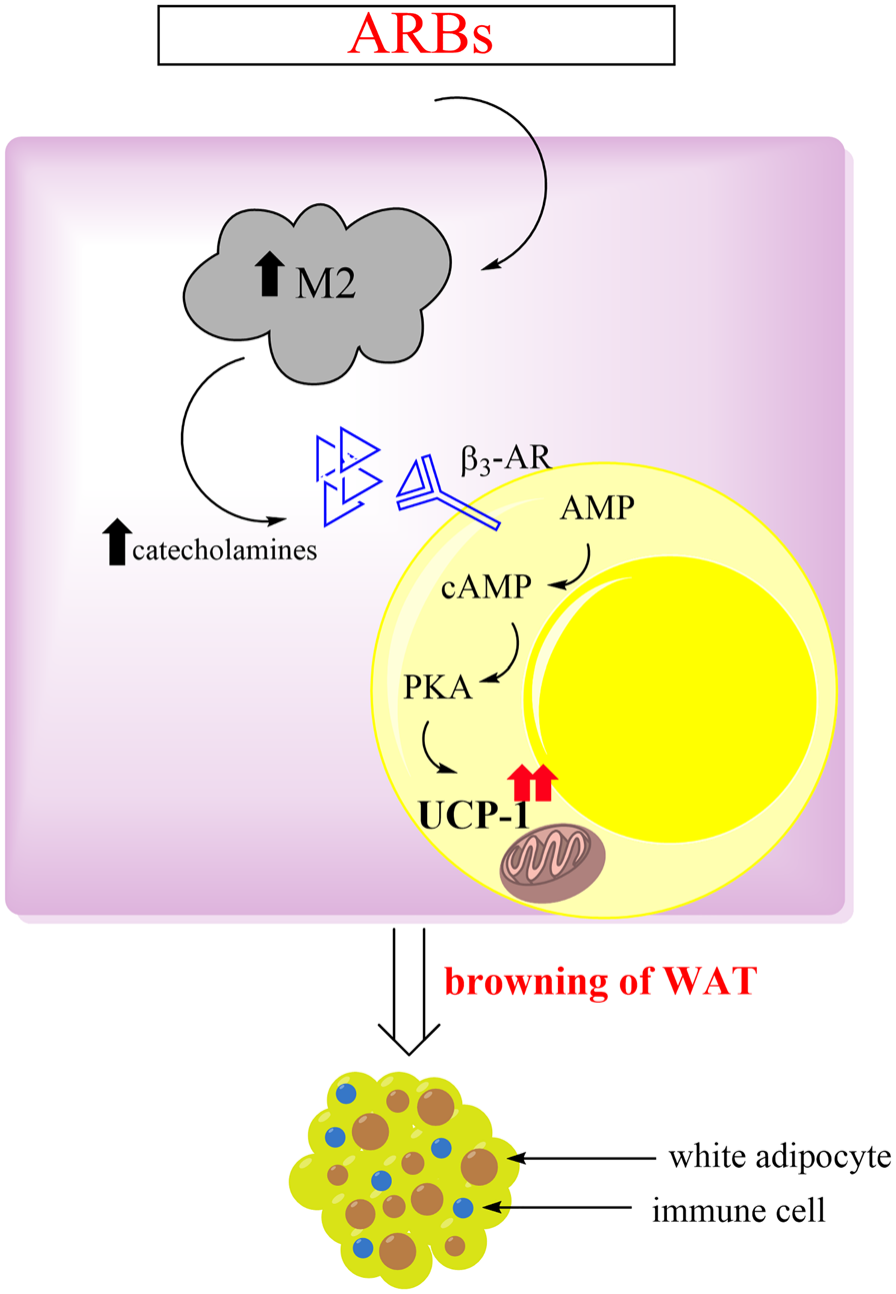
Suggested pathway by which ARBs awoke browning. *ARBs* selective and competitive type 1 angiotensin II receptor (AT-1) blockers; *M2* macrophage type 2, *β3-AR* β3-adrenergic receptor, *AMP* adenosine monophosphate, *cAMP* cyclic adenosine monophosphate, *PKA* protein kinase A, *UCP-1* uncoupling protein-1

In addition, in another study authors claimed that in the basis of metabolic diseases is infiltration of adipose tissue with particularly M1 macrophage population. This is proinflammatory subpopulation of macrophages which induce insulin resistance and chronic inflammation in tissue. Oppositely, M2 macrophages are alternatively activated and inhibit obesity induced inflammation. Several signalling pathways can upregulate M2 polarization including PPARγ and peroxisome proliferator-activated receptor delta (PPARδ) as key transcription factors [125, 132]. It was reported that telmisartan can promote M2 polarization via activation of PPARγ and evokes browning of white fat cells [133]. The significance of crosstalk between telmisartan and PPARγ in upregulation of M2 macrophages is confirmed since other AT-1 blockers without PPARγ agonistic activity did not activate M2 polarization. Therefore, the polarizing effect of telmisartan is due to its ability to activate PPARγ, not to antagonistic effect on AT-1 receptors. Notably, among many secretary molecules that are released from telmisartan-activated macrophages, catecholamines appear as very important ones since they are well-known stimulators of thermogenesis via activation of β3 adrenergic receptor and cAMP-PKA axis. As a final mediator in this cascade, PKA directly activates mTORC1 to drive adipose browning (Fig. 4) [125, 134, 135].

A significant reduction in damaging effects of high-fat intake was reported after treatment with valsartan. Beside favorable results in glucose metabolism, valsartan decreased signs of visceral adipose tissue inflammation as well as infiltration of macrophages in white adipocytes. The expression of proinflammatory markers, that are in the basis of obesity induced inflammation, such as TNFα, IL-6, IL-12, interferon gamma (IFNγ) and monocyte chemotactic protein-1 (MCP-1) were controlled with valsartan as well as adequate levels of adiponectin in adipocytes. Moreover, in overweight mice valsartan led to less adipocyte size which is in consistent with observation that antagonists of AT-1 receptors enhance function of adipocyte and stimulate formation of smaller and more metabolically active adipocytes [136]. This preclinical result could be supported with findings from large clinical study indicating that 26-weeks valsartan treatment significantly reduced adipocyte size in abdominal subcutaneous adipose tissue which is the consequence of decreased macrophage infiltration in fat cells depots. Indeed, improvement in adipose tissue function after valsartan treatment affects the higher insulin sensitivity thus preventing development of metabolic disturbance in high-risk patients [137].

The previous studies focused on examining the effects of separately treatment with AT-1 antagonists or NPs on adipose tissue metabolism and their beneficial effects were listed above. Nonetheless, Jordan et al. investigated the effects of combined sacubitril/valsartan treatment in hypertensive patients with obesity, indicating remarkably improved insulin sensitivity and increased lipolysis of abdominal subcutaneous adipose tissue but did not affect whole-body lipolysis [138]. In order to clarify a marked improvement in insulin sensitivity the same group of authors examined the effects of combined sacubitril/valsartan treatment on abdominal adipose tissue phenotype in hypertensive patients with obesity. Studies mentioned that sacubitril/valsartan did not induce changes in expression of genes that are involved in lipolysis, nor this treatment led to remarkably increase in transcriptional change in abdominal subcutaneous fat cells depots. Moreover, the expression of proteins that are included in mitochondrial oxidative pathway or NPs signalling pathway was not detected [139]. Furthermore, it was reported that lower NPR-A:NPR-C ratio might lead to increased NPRs clearance and lower biological activity of NPs in adipose tissue of obese hypertensive patients compared to normotensive lean individuals, leading to reduce AMP-signalling pathways. Together, this fact implies that preserved NPs levels and NPRs activity may be important in obese hypertensive individuals. Moreover, due to adipocytes are target cells for NPs different adipose mass in obese patients might affect NPs plasma level and thus take influence on biological activity on NPs [140]. This finding may be a reasonable explanation for inconsistent results from this study with those from experimental research due to slight metabolic phenotype alteration in subcutaneous adipose tissue after combined sacubitril/valsartan treatment. Given that this clinical study investigated effects of sacubitril/valsartan only on subcutaneous adipose tissue depots it still remains unknown whether these drugs would induce phenotype changes in other adipose tissue depots.

## Conclusion

Recent focused investigations have provided a new insight into the origins of BAT, as well as into its activity and metabolism in humans. As adipose tissue is closely involved in almost all interaction with cardiovascular system including its important role in cardiovascular pathogenesis, the discovery of agents that promote browning of WAT has opened a new field for development of new approaches to combat with obesity and prevent T2DM and metabolic syndrome occurrences. Although the list of novel browning promoters is expanded, new agents are still being sought that will not only raise BAT activity but also stimulate brown adipocytes appearances in locations that are typical for WAT.

Cold is well known stimulus for BAT activation which triggers its thermogenic activity, enhances energy expenditure in BAT and increases glucose uptake and insulin sensitivity. To boost the circulating levels of NPs, sacubitril regulates glucose metabolism and insulin resistance, but also increases the postprandial lipid oxidation contributing to energy expenditure. Moreover, this drug promotes lipolysis in adipocytes and reduces body weight. As obesity is firmly linked to increased plasma RAS components levels, valsartan as AT-1 blocker has strong potential to gain weight loss, reduce glycemic level and enhance the expression of thermogenic genes.

Due to the pronounced effects of cold and sacubitril/valsartan treatment on function and metabolism of BAT, the primary goal of further research should be to investigate the synergistic effects of the combined use of sacubitril and valsartan at low temperature environmental conditions. The principal overlap between these three browning strategies exists and it is primarily related to mTORC1 activation. Although all of these three stimuli activate mTORC1 via different signalling pathways, their common effect is related to enhanced expression of thermogenic genes contributing to formation of beige adipose tissue. Therefore, these results will distribute the knowledge in a new field of research both driving an interest in the area of brown adipocytes function, but also giving the ability to use the full potential of BAT as a therapeutic strategy in metabolic diseases.

## Data Availability

Not applicable.

## Abbreviations

12,13-diHOME: 12,13-Dihydroxy-9Z-octadecenoic acid
AMPK: 5’-AMP-activated protein kinase
ANP: Atrial natriuretic peptide
ARBs: Type 1 angiotensin II receptor blockers
AT-1: Type 1 angiotensin II receptor
ATF2: Nuclear factor activating transcription factor 2
ATP: Adenosine triphosphate
BAT: Brown adipose tissue
BNP: Brain natriuretic peptide
C/EBPα: CCAAT/enhancer-binding protein α
cAMP: Cyclic adenosine monophosphate
cGMP: Cyclic guanosine monophosphate
CNP: C-type natriuretic peptide
CRE: CAMP-response element
CREB: CAMP-response element binding protein
DIO2: Type 2 iodothyronine deiodinase
DPP-4: Dipeptidyl peptidase-4
EAT: Epicardial adipose tissue
FFA: Free fatty acids
FGF: Fibroblast growth factor
GLUT4: Glucose transporter protein 4
HFD: High fat diet
HSL: Hormone-sensitive lipase
IFNγ: Interferon gamma
IL: Interleukin
MCP-1: Monocyte chemotactic protein-1
mLST-8: Mammalian lethal with Sec13 protein 8
mTOR: Mammalian target of rapamycin
NF-κβ: Nuclear factor kappa β
NPRs: Natriuretic peptide receptors
NPR-A: Natriuretic peptide receptor-A
NPs: Natriuretic peptides
p38-MAPK: P38-mitogen-activated protein kinase
PGC-1α: PPARγ-coactivator 1-α
PGE_2_: Prostaglandin E_2_
PKA: Protein kinase A
PKG: Protein kinase G
PPARα: Peroxisome proliferator-activated receptor alpha
PPARγ: Peroxisome proliferator-activated receptor gamma
PPARδ: Peroxisome proliferator-activated receptor delta
PRDM-16: PR domain zinc finger protein 16
RAPTOR: Regulatory associated protein of mTOR
RAS: Rennin-angiotensin system
RIP140: Receptor-interacting protein 140
RXR: Retinoid X receptor
SAT: Subcutaneous adipose tissue
sEHs: Soluble epoxide hydrolases
SGLT-2: Selective sodium-glucose cotransporters-2
SIRT1: NAD-dependent protein deacetylase sirtuin 1
T2DM: Type 2 diabetes mellitus
T3: Tri-iodothyronine
T4: Tetraiodothyronine
TLE3: Transducin-like enhancer of split 3
TNFα: Tumor necrosis factor alpha
TZDs: Thiazolidinediones
UCP-1: Uncoupling protein-1
VAT: Visceral adipose tissue
VDR: Vitamin D receptor
WAT: White adipose tissue
β3-AR: β3-Adrenergic receptor
